# The expanding role of microRNAs in the biology and control of veterinary parasitic nematodes

**DOI:** 10.3389/fvets.2025.1668932

**Published:** 2025-09-19

**Authors:** Vishnu Manikantan, Andrea Pires dos Santos, Sriveny Dangoudoubiyam

**Affiliations:** Department of Comparative Pathobiology, College of Veterinary Medicine, Purdue University, West Lafayette, IN, United States

**Keywords:** miRNAs, nematodes, parasites, biomarkers, anthelmintic resistance, immunomodulation, extracellular vesicles

## Abstract

Parasitic nematodes threaten animal health globally, contributing to substantial losses in livestock productivity and posing zoonotic risks through infections in companion animals. There is a growing concern over widespread resistance to anthelmintic drugs, necessitating new molecular approaches for parasite control. MicroRNAs (miRNAs) are small non-coding RNAs that regulate gene expression and have emerged as key modulators of nematode development, growth, stage transitions, host-pathogen interactions, and parasite survival. Certain miRNAs are expressed in a stage- and sex-specific manner, and many are selectively secreted via extracellular vesicles, enabling direct interactions with the host. The detection of worm-derived miRNAs in blood of an infected host highlights their potential as early diagnostic biomarkers for nematode infections. Emerging evidence links miRNAs to anthelmintic resistance. This review provides an overview of miRNA biogenesis, gene regulation mechanisms, and current miRNA discovery and experimental validation approaches. Importantly, it highlights species-specific advances in miRNA research in parasitic nematode infections of veterinary importance, emphasizing their roles in parasite biology, immune modulation, and drug resistance.

## Introduction

Parasitic nematode (worm) infections in livestock, companion animals, and wildlife are a major concern in Veterinary Medicine. In livestock, these infections threaten health and productivity, resulting in significant economic losses. Trichostrongylid nematodes like *Haemonchus contortus, Ostertagia ostertagi*, and *Cooperia* spp. are particularly prevalent in grazing animals such as cattle, sheep, and goat, and are responsible for widespread production losses (1, 2). In general, parasitism in livestock can lead to decreased feed intake, interference with digestion and nutrient uptake, reduced growth rate, weight gain, and productivity (3). Additionally, body resources diverted to immune functions can affect behavior and reproductive performance (4). In the United States alone, the economic losses due to gastrointestinal nematodiasis in beef cattle are estimated at ~$8.5 billion annually (5). Globally, the economic impact of nematode infections in livestock is further exacerbated by rising drug resistance among these parasites. Resistance to widely used anthelmintic drugs has been recorded in nematode populations across continents, diminishing the effectiveness of standard treatments (6). This growing problem highlights the urgent need for new management techniques, including treatments grounded in a molecular understanding of nematode biology.

In companion animals and wildlife, nematode infections directly impact animal health, and some species also pose significant zoonotic risks (7). Companion animals, such as dogs and cats, are vulnerable to infections by various nematodes: *Toxocara* spp., *Ancylostoma* spp., *Dirofilaria immitis*, and *Strongyloides* spp. are some that can lead to acute or chronic health issues such as malnutrition, anemia, or cardiopulmonary complications (8). The financial burden of frequent veterinary consultations, diagnosis, and treatment is substantial (9). Extended treatment plans for certain infections can further add to the economic strain for pet owners managing these conditions. These infections can also negatively affect animal behavior and strain the human-animal bond (10, 11). Like the livestock parasites, there is growing concern about anthelmintic resistance in nematodes infecting companion animals. The most recent example of this is the rise and expansion of single and multi-drug-resistant hookworm infections in dogs across the United States (12).

Unlike bacteria or viruses, parasitic nematodes do not replicate within their hosts. Instead, they undergo complex developmental stage transitions from immature larvae to mature adult forms that reproduce within their host. This happens over a prolonged period (days to months), leading to extensive interaction between the host and parasites. During this interplay, the parasite manipulates the host environment and pathways to prevent parasite expulsion and preserve host fitness to support parasite survival. Understanding the mechanisms underlying the host-pathogen interactions is essential for developing effective diagnostic and therapeutic tools.

Among the various mechanisms through which nematodes interact with their hosts, miRNAs have emerged as key regulators of gene expression, playing crucial roles in the parasite's lifecycle and host-parasite interactions (13–15). The first miRNA, lin-4, was discovered in 1993 by Victor Ambros and colleagues in the nematode *Caenorhabditis elegans* (16), and the first extensive exploration of miRNAs in parasitic nematodes was performed in the filarial nematode *Brugia malayi* in 2010 (17). Since then, due to significant advancements in nematode genomics and high-throughput deep sequencing technologies, numerous miRNAs have been identified from parasitic nematodes belonging to different clades (Table 1). These molecules could offer valuable insights into the molecular mechanisms of nematode interaction with the host and serve to develop novel diagnostic tools and potential therapeutic interventions.

**Table 1:** Summary of miRNA studies in parasitic nematodes across developmental stages and sample types.

**Organism**	**Clade**	**Stage/sample**	**No. of miRNAs identified**	**Ref. genome of miRNA**	**Reference**
*Ascaris suum*	III	Germline, zygote, embryo L1, L2	97	*Ascaris suum*	(115)
		Female and male adults	494 and 505	*Ascaris suum*	(57)
		Female adults	497	*Ascaris suum*	(111)
		L3, L4 and adult worm–EVs	51, 40, and 29	*Ascaris suum*	(55)
*Ascaris lumbricoides*	III	Female adults	171	*Ascaris suum*	(111)
*Toxocara canis*	III	Female and male adults	619 and 560	*Toxocara canis*	(103)
*Anisakis pegreffii*	III	L3 and L4 EV	126 and 30	*Anisakis simplex*	(40)
*Brugia malayi*	III	Female and male adults, mf	145	*Brugia malayi*	(119)
		L3 and L3 EV	88 and 52	*Brugia malayi*	(120)
		mf EVs and mf secreted products	130	*Brugia malayi*	(80)
*Brugia pahangi*	III	L3 and mixed-sex adult	75 and 76	*Brugia malayi*	(113)
		L3	NA	*Brugia malayi*	(56)
*Dirofilaria immitis*	III	Mixed-sex adult worms	1,063	*Brugia malayi*	(94)
		Culture media–L3, L4, mf, female and male adults	129, 77, 55, 83, and 71	*Dirofilaria immitis*	(97)
		Culture media- L3 and L4	187	*Dirofilaria immitis*	(98)
*Hemonchus contortus*	V	L3 and adult	89 and 109	*Hemonchus contortus*	(113)
		Female adults gut	96	*Hemonchus contortus*	(128)
		L3, L4, male and female adults, gut	NA	*Hemonchus contortus*	(13)
		L4 EV, adults EV	NA	*Hemonchus contortus*	(79)
		L3, xL3 and L4	86, 87, and 84	*Hemonchus contortus*	(114)
*Teladorsagia circumcincta*	V	L4 ES	47	miRBase	(79, 114)
*Angiostrongylus cantonensis*	V	L3 and L4	526 and 376	*C. elegans, B. malayi, H. sapiens, M. musculus, and R. norvegicus*	(129)
		Female and male adults	134 and 196	*C. elegans*	(130)
		Young adults	252	miRBase	(131)
		L5, L5 ES, and adult ES	65, 52, and 39	*Angiostrongylus cantonensis*	(132)
*Trichinella spiralis*	I	Muscle larvae	130	*C. elegans, B. malayi*	(133)
		Muscle larvae, adult worms and newborn larvae	250	*Trichinella spiralis*	(134)
		Muscle larvae, intestinal infective larvae	2,000	*Trichinella spiralis*	(135)
		Adult EVs	NA	*Trichinella spiralis*	(89)
		Newborn larvae and EV	183 and 117	*Trichinella spiralis*	(136)
		Larvae-derived EVs	64	*Trichinella spiralis*	(90)
		Larvae exosomes	NA	*Trichinella spiralis*	(92)
*Trichuris suis*	I	L1/L2, L3, L4, female and male adults, stichosomal and non-stichosomal portions of adults	319	*Trichuris suis*	(137)
*Strongyloides stercoralis*	IV	L1 and iL3	385	*Strongyloides stercoralis*	(125)
		iL3, female adults	265	*Strongyloides stercoralis*	(126)

NA, not available.

This review discusses the general aspects of miRNA biogenesis, the mechanism of gene regulation, and the current miRNA discovery and validation strategies. It highlights the species-specific advances in miRNA research in parasitic nematodes of veterinary importance, emphasizing their roles in parasite biology, host immune modulation, and drug resistance.

## Canonical and non-canonical miRNA biogenesis

MicroRNAs are small, single-stranded, non-coding RNAs, typically 18–22 nucleotides long, that regulate gene expression at the post-transcriptional level. They function by binding to complementary sequences on messenger RNA (mRNA), leading to either mRNA degradation or translational repression (18). MicroRNA biogenesis is a multistep process that begins with transcription in the nucleus and culminates in the formation of mature miRNA in the cytoplasm. Approximately half of all known miRNAs are intragenic, primarily located within introns and less commonly in exons of protein-coding genes, and they are often co-transcribed with their host genes. The remaining miRNAs are intergenic, transcribed independently under their own promoters (19, 20). MicroRNAs are produced through canonical and non-canonical pathways, with the canonical pathway being the primary route. Within this pathway, RNA polymerase II transcribes a primary-miRNA (pri-miRNA) transcript from genomic DNA. These are processed by the microprocessor complex, consisting of the ribonuclease III enzyme, Drosha, and the double-stranded RNA binding protein DiGeorge Syndrome Critical Region 8 (DGCR8), as well as accessory factors, cleaving the pri-miRNA to an ~80-nucleotide hairpin pre-miRNA (21). The pre-miRNAs are then transported to the cytoplasm via the Exportin-5 complex, where they are further cleaved by the RNase III endonuclease Dicer, which cleaves the terminal loop to form a mature miRNA duplex of 18–22 nucleotides (22, 23). Finally, either the 5p or 3p strand of the mature miRNA duplex is integrated into the Argonaute (AGO) protein family, creating a miRNA-induced silencing complex (miRISC) (24, 25). The non-canonical miRNA biogenesis includes Drosha/DGCR8-independent and Dicer-independent pathways. Pre-miRNAs, such as mirtrons, which are produced from introns during mRNA splicing, belong to the Drosha/DGCR8-independent pathway (20, 26). In the Dicer-independent pathway, endogenous short hairpin RNAs (shRNAs) are processed by Drosha and exported to the cytoplasm through Exportin 5/RanGTP. These shRNAs are then cleaved by AGO-2 in a Dicer-independent manner to form the mature miRNA (27).

## Mechanism of action of microRNAs

MicroRNAs regulate gene expression primarily by inhibiting translation initiation or promoting the degradation of target mRNA. To achieve target specificity, the 2–8 nucleotide seed region of mature miRNA, within the miRISC complex, will interact with complementary sequences known as miRNA response elements (MREs), typically located in the 3′ untranslated region (3′ UTR) of target mRNAs (28). Alternatively, miRNAs can also bind to 5′ UTR, coding sequences, and promoter regions, which can either repress or activate translation depending on specific mRNA and cellular conditions (29–31). The degree of complementarity between the seed sequence and the MRE determines whether the target mRNA undergoes AGO-2-mediated slicing or miRISC-induced translational repression and mRNA decay (32, 33). In cases of perfect or near-perfect complementarity, AGO2 exhibits endonuclease activity that cleaves the target mRNA, leading to miRNA destabilization and degradation (34). Most miRNA interactions in animal cells are not perfectly complementary. In such cases, mismatches prevent AGO2-mediated slicing, resulting in translational inhibition and mRNA decay through non-endonucleolytic mechanisms (AGO1, 3 and 4) (33).

**Figure 1:**
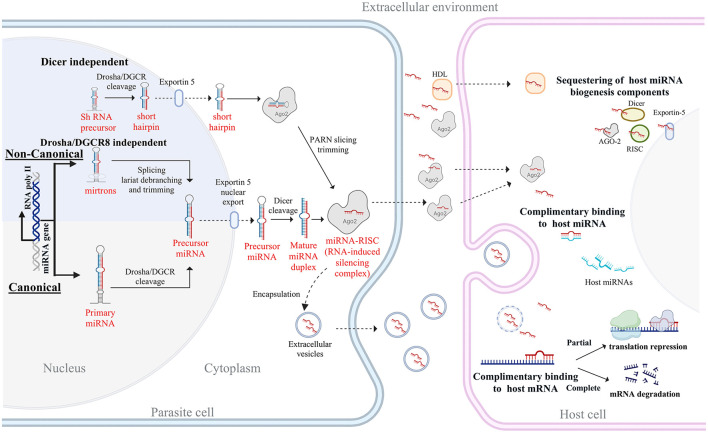
Overview of miRNA biogenesis and host-targeted actions in parasitic nematodes. The diagram illustrates the biogenesis of miRNAs through both canonical and non-canonical pathways. In the canonical pathway, primary miRNAs (pri-miRNAs) are transcribed by RNA polymerase II and processed by the Drosha-DGCR8 complex into precursor miRNAs (pre-miRNAs), which are then exported to the cytoplasm via Exportin-5. Dicer cleaves pre-miRNAs into mature miRNA duplexes, which are incorporated into the RNA-induced silencing complex (RISC) with Argonaute (Ago2). The non-canonical pathways include mirtron-derived miRNAs that bypass Drosha processing and shRNA-derived miRNAs that are processed independently of Dicer. Parasite-derived miRNAs can be released into the extracellular environment via extracellular vesicles or bound to proteins such as Ago2 and high-density lipoproteins (HDL). Upon uptake by the host cell, these miRNAs can modulate host gene expression through sequestration of host miRNA machinery, direct binding to host miRNAs, or targeting host mRNAs. Partial complementarity leads to translation repression, while complete binding results in mRNA degradation, ultimately influencing host immune responses and facilitating parasite survival.

While the core mechanisms of miRNA biogenesis and action are similar between mammals and nematodes, there are also notable differences, especially in the number and functions of the Dicer and Argonaute proteins (35). In mammals, a single Dicer protein (DICER1) processes both miRNAs and siRNAs, whereas nematodes such as *Caenorhabditis elegans* possess multiple Dicer proteins, each specialized for distinct small RNA pathways (36). Similarly, in nematodes, the Argonaute family has undergone extensive diversification, giving rise to specialized clades such as the WAGO (Worm-specific Argonautes), which are largely absent in mammals (37). Mammals rely on a more limited set of Argonautes, with AGO2 being the primary slicer-active protein, reflecting a more streamlined but less diversified RNAi machinery (35). Beyond these molecular distinctions, nematode miRNAs are highly stable and developmentally timed to facilitate stage transition in the life cycle of the parasite (38, 39). Remarkably, parasite-derived miRNAs can regulate host miRNAs either by sequestering host miRNA biogenesis components such as AGO and Dicer or by binding to host miRNAs via perfect or near-perfect complementary base pairing (40–42). These mechanisms allow parasite miRNAs to manipulate both host mRNAs and miRNAs, finely modulating host gene expression to eventually promote a permissive environment for parasite survival and the establishment of chronic infections (43, 44) (Figure 1).

## MicroRNA identification, target prediction, and experimental validation

MicroRNAs can be directly identified from high-throughput sequencing data by mapping reads to reference genomes. Computational tools such as miRDeep (45), miRExpress (46), miRanalyzer (47), and mireap (http://sourceforge.net/projects/mireap/) are used to predict known and novel miRNAs by specifically detecting characteristic features of miRNA sequences, including hairpin structure and sequence conservation across related species (48, 49). To understand the biological roles of these miRNAs, it is important to identify their target genes. Computational tools such as miRanda (50), RNAhybrid (51), PITA (52), TargetScan (53), and PicTar (54) are widely used for miRNA target prediction. These tools apply various scoring criteria for each miRNA-target prediction, including sequence complementarity, the evolutionary conservation of target sites, target accessibility, and the free energy of binding (49). However, these algorithms were mainly designed for human or model organism miRNAs and are of limited utility for miRNA target prediction in parasitic helminths. The accuracy of these target gene predictions is often questionable for various reasons, such as the extensive diversity among nematodes, incomplete annotated genomes, and the lack of accurate 3′ UTR sequence information. Additionally, a lack of consideration for evolutionary conservation and stage-specific variations in miRNA expression constrains specific target prediction. Despite these limitations, these algorithms have been used either alone or in combination for miRNA target prediction in parasitic nematodes (13, 55–57). This results in significantly different results depending on the tool or the parameter used, necessitating cautious interpretation of the results (48, 58).

Computational predictions of miRNA targets require experimental validation to confirm their biological significance. The experiments conducted typically address one or more of the following criteria: (1) direct binding between miRNA and target mRNA, demonstrated using luciferase reporter assays or immunoprecipitation techniques, (2) co-expression of the miRNA and its target mRNA, assessed through RT-qPCR, RNA-seq, or Northern blotting, (3) regulatory effects on mRNA or protein levels, measured by RT-qPCR, Western blotting, or mass spectrometry and (4) specificity, confirmed using miRNA inhibitors or mimics [reviewed in (59)]. The experimental methods used in assessing the functional miRNA–mRNA interactions are briefly described.

Luciferase reporter assays are among the most common methods for studying miRNA–mRNA interactions. In this method, the 3′ UTR of the target gene is inserted downstream of a luciferase gene in a reporter plasmid and co-transfected into cells along with the miRNA of interest. If the miRNA successfully binds to the 3′ UTR, it leads to degradation or translational repression of the luciferase mRNA, ultimately reducing luciferase protein levels. The corresponding drop in luminescence provides a quantifiable measure of miRNA activity (13, 56, 60). HITS-CLIP (High-throughput sequencing of RNA isolated by crosslinking immunoprecipitation) is a powerful tool for identifying miRNA–mRNA interactions and relies on UV irradiation to cross-link miRNAs and their target mRNAs to Argonaute and other RISC proteins. Once cross-linked, the RISC complexes are isolated through immunoprecipitation using specific antibodies, and the sequences are subsequently analyzed to determine the target miRNA binding regions (61, 62).

RT-qPCR can be utilized to analyze total RNA extract, employing specific primers or probes for both miRNA and its corresponding mRNA target to determine whether they are expressed concurrently. RNA-seq provides a genome-wide view of miRNA and mRNA co-expression, while Northern blotting can confirm size and expression levels of specific miRNAs. However, changes in mRNA levels measured by these techniques do not always directly reflect miRNA activity, since miRNAs can also inhibit translation without inducing mRNA degradation (63). Assessment of the impact of miRNA determined by changes in protein abundance provides the most direct evidence of functional repression, and it can be carried out using Western blot assays or mass spectrometry.

To assess specificity, researchers use miRNA inhibitors and miRNA mimics (64). MicroRNA inhibitors are chemically modified antisense oligonucleotides (anti-miRs or antagomiRs) that bind to the miRNA and prevent it from interacting with its target, leading to restoration of expression of the target gene if the interaction is specific. Conversely, miRNA mimics are synthetic double-stranded RNA molecules that resemble endogenous miRNAs, enhancing their function and leading to further suppression of the target gene (64). These assays use high concentrations of synthetic precursors that can saturate the RISC complex and compete with endogenous miRNAs, thus increasing the risk of false-positive results (65). We can determine whether changes are miRNA-dependent or not by comparing gene transcript levels or protein abundance in cells treated with either miRNA inhibitors or mimics. Inhibiting a miRNA is generally preferred, but achieving potent miRNA inhibition can be challenging, making overexpression a more commonly used approach despite its potential drawback of non-physiological interactions that would not typically occur in cells (66).

## MiRNAs in nematode development, stress response, and reproduction

Parasitic nematodes have adapted to changing environments, as evidenced by their complex life cycles involving infective stages in the environment or intermediate host, and adult stages in the definitive host. The transition between various life stages is orchestrated by complex regulatory networks, including miRNA-mediated pathways (67, 68). MicroRNAs play a major role by fine-tuning the gene expression profiles to promote molting, growth, and differentiation in a specific ecological niche (68–70). They can also influence the nematodes' ability to respond to different environmental stresses, including temperature fluctuations, oxidative and osmotic stress (71, 72). Initially discovered in *C. elegans* and later extended to parasitic nematodes, certain miRNAs are pivotal for developmental transitions between lifecycle stages. For instance, let-7 and miR-36 families regulate key developmental phases, such as embryogenesis and larval development in *C. elegans* as well as in parasitic nematodes like *Brugia pahangi* and *Ascaris suum* (56, 73, 74). In *Haemonchus contortus*, hco-miR-228 and hco-miR-235 were significantly enriched in the infective L3 stage and function to maintain larval arrest, likely by reinforcing DAF-16/FOXO activity and suppressing metabolic pathways needed for development (13). These findings highlight how miRNAs fine-tune gene expression to adapt to various environmental conditions and life cycle stages.

In addition to stage-specific roles, miRNAs also show sex-biased expression in parasitic nematodes, influencing sex-specific gene regulation. For example, in *Brugia malayi*, miRNAs like the bma-let-7 family members and bma-miR-2 variants are more highly expressed in males, while bma-miR-36 family members are predominant in females (17). These sex-specific miRNAs regulate key reproductive genes, such as those involved in sperm motility and ovarian functions (75). Similar patterns of sex-biased miRNA expression have been observed in *A. suum*, where distinct sets of miRNAs target reproductive proteins in males and females (57). This intricate regulation by miRNAs underscores their importance in the reproductive success and developmental plasticity of parasitic nematodes. Beyond their roles in reproduction and development, miRNAs also contribute to parasite survival by modulating the host immune response (14, 76, 77). These diverse functions of miRNAs will be discussed in later sections, with examples from different parasitic nematode species.

## miRNA-mediated host-nematode cross-talk

Since their discovery in free-living nematodes and later expanding to parasitic species, including nematodes, miRNAs have emerged as critical regulators of gene expression, involved in parasite development, survival, and host interactions. MicroRNAs have been studied in various life cycle stages, i.e., infective larvae, migratory larvae from the host tissues, and adults (males and females). Additionally, miRNAs secreted into the culture medium (*in vitro*) either as a component of the parasite's excretory-secretory products (vesicle-free form) or packaged into extracellular vesicles (EVs), particularly exosomes, have been studied. Exosomes are small, membrane-bound vesicles ~30 to 100 nm in diameter capable of transporting DNA, RNA, and proteins. The secretion of miRNAs through exosomes can modulate host-parasite interactions by regulating gene expression in the parasite and the host (43, 78). Evidence suggests that the secretion and loading of at least some helminth miRNAs into EVs is not arbitrary but a selective and regulated event. For example, the miRNA composition of helminth-derived EVs often differs from the miRNA expression profile observed within the parasite's cells (55, 57, 79, 80). Notably, miRNAs that are abundant inside the parasite cells are often underrepresented or absent in secreted EVs, suggesting a selective sorting mechanism of miRNA cargos. This selectivity seems to have been evolutionarily conserved within different species of helminths, with certain miRNAs being consistently detected in EVs, suggesting that these miRNAs are necessary for host–parasite communication. Several secreted miRNAs have been investigated for their potential use as biomarkers of nematode infection, particularly for species that are tissue-residing or present in the host circulation, where parasite-derived miRNAs are more likely to be detected in host biofluids. Table 2 lists some of the microRNAs identified and evaluated as biomarkers in various parasitic nematodes. Comparative genomics has shown that many miRNAs are conserved not only across nematode species but also among distantly related taxa (81), highlighting their essential roles in gene regulation and the maintenance of key biological functions. Interestingly, several miRNAs from parasitic nematodes share striking sequence identity with mammalian miRNAs, further suggesting that these molecules may perform similar regulatory functions across species.

**Table 2:** MicroRNAs investigated as biomarkers of nematode infections.

**Organism**	**Clade**	**Host**	**Sample**	**Circulatory miRNAs investigated as biomarkers in the host**	**Origin of miRNA**	**Reference**
*Dirofilaria immitis*	III	Dog	Plasma	miR-100a/d/c, miR-71, miR-34, miR-228, miR-50, and miR-57	Parasite	(96)
	III	Dog	Plasma	let-7, miR-92, miR-71, miR-100b/d, miR-81, miR-1	Parasite	(95)
*Brugia pahangi*	III	Dog	Plasma	miR-71 and miR-34, miR-223	Parasite	(96)
*Onchocerca volvulus*	III	Human	Serum	miR-71 and miR-34	Parasite	(96)
	III	Human	Plasma	miR-71 and miR-100a/d, bantam a, Lin-4, miR-87-3p	Parasite	(138)
*Onchocerca ochengi*	III	Cow	Nodule fluid	miR-71, bantam–a/b/c, miR-100a/c/d miR-86-5p	Parasite	(138)
	III	Cow	Plasma	miR-100a, miR-81	Parasite	(139)
*Loa loa*	III	Baboon	Plasma	miR-36, miR-92, lin-4, miR-100a/d	Parasite	(139)
*Angiostrongylus cantonensis*	V	Human	Serum	miR-146a	Parasite	(140)
*Toxocara canis*	III	Human	Plasma	miR-21 and miR-103a	Host	(104)
*Trichinella spiralis*	I	Mouse	Serum	miR-467a-3p, miR-467d-3p, miR-376b-3p, miR-664-3p, miR-292a-5p	Host	(141)
*Trichuris suis*	I	Pig	Serum	let-7d-3p	Host	(142)

## MicroRNAs and drug resistance in nematodes

Dealing with the parasitic nematodes in livestock has become increasingly challenging due to resistance issues that have arisen over time. Three principal anthelmintic categories dominate livestock care: benzimidazoles, cholinergic agents like levamisole, and macrocyclic lactones, such as ivermectin (IVM). Alarmingly, there have been reports of resistance emerging throughout the world against all three categories of treatments, threatening both the sustainability of livestock farming and global food security (82–85). Among several factors, alterations in miRNA can lead to shifts in the expression of resistance-related genes, resulting in drug resistance. Recent studies using microarrays have shed light on a link between miRNA expression and resistance to IVM. For instance, the miRNA hco-miR-9551 of *H. contortus* showed upregulation in four IVM-resistant strains compared to their susceptible counterparts (86). It's interesting to note that a comparable increase in miRNA-9551 has also been noticed in multi-drug resistant *Teladorsagia circumcincta*, a gastrointestinal nematode of small ruminants (86, 87); miR-9551 likely targets CHAC1 domain-containing proteins, which exhibit gamma-glutamyl cyclotransferase (GGCT) activity, crucial for glutathione degradation. A reduction in CHAC1 protein levels could lead to increased glutathione levels, which in turn may augment the detoxification processes or protect against apoptosis in drug-resistant worms. Another example is the negative regulation of the expression of nicotinic acetylcholine receptor (nAChR) subunits UNC-29 and UNC-63 by cel-miR-1 in *C. elegans*. In miR-1 mutants, increased levels of these subunits showed reduced sensitivity to the drugs acetylcholine and levamisole, likely due to altered receptor subunit composition affecting biogenesis or function (88). These discoveries reveal the roles of miRNAs in controlling gene expression associated with drug resistance in parasitic nematodes. In this latter section, we discuss the status of miRNA research in parasites of veterinary importance.

## Trichinella spiralis

*Trichinella spiralis* is a zoonotic nematode acquired by ingesting raw or undercooked meat containing infective larvae. It exhibits a unique lifestyle with adults in the intestinal tract and larvae nestled within modified skeletal muscle fibers called nurse cells. Trichinellosis is characterized by gastrointestinal symptoms followed by muscle pain, fever, and periorbital edema during larval migration in humans. Interestingly, secreted miRNAs and those packaged into EVs have been described in *T. spiralis* that modulate the host response in the parasite's favor (89, 90). *Trichinella* infection triggers the remodeling of damaged muscle cells into nurse cells instead of typical regeneration and repair (91), and a miRNA, miR-31 homolog, secreted by *T. spiralis* larvae has been implicated (89) to suppress the myogenic program vital for the differentiation and repair of muscles. Additionally, at least three miRNAs, tsp-miR-153, tsp-miR-1-3p, and tsp-let-7-5p, identified in *T. spiralis* larval EVs may be responsible for modulating the host immune defense. The miRNA, tsp-miR-153, targets the bcl2 gene of host intestinal epithelial cells with resultant apoptosis (92) and impaired intestinal barrier function, crucial for maintaining host defense against pathogens. Whereas tsp-miR-1-3p and tsp-let-7-5p facilitate alternate activation of macrophages and release of anti-inflammatory cytokines. They also inhibit the activation of host fibroblasts and collagen, thus preventing calcification and supporting the chronic persistence of *T. spiralis* larvae (90). These examples establish the capability of parasite miRNAs to alter the host cellular processes to benefit their survival significantly.

## Dirofilaria immitis

The filarid, *Dirofilaria immitis*, is commonly known as the heartworm of dogs and cats. After an infective mosquito releases *D. immitis* L3 developmental stages into the host, progression of these stages to develop into adult males and females occurs, which reside in the pulmonary artery and the right ventricles of the heart. Several studies have been undertaken to discover biomarkers for the early detection of this infection (93–98). Although the ranking of the individual miRNAs differs, there is agreement on the miRNAs identified in the top 10 (97, 98). With its initial discovery as a predominant miRNA isolated from mixed-sex adult worms obtained from a dog at necropsy (94), dim-miR-71 has also been detected in infected dog plasma (96). Despite its lower levels, a second miRNA candidate identified in multiple studies is dim-miR-34 (94, 96). Identifying circulating parasite-derived miRNAs in biofluids of an infected animal is significant, but dim-miR-71 and dim-miR-34 seem to be present in all developmental stages of *D. immitis* and cannot necessarily help with early diagnosis. Additionally, the levels of these two miRNAs in infected dogs did not correlate with the burden of infection (93). In regions where both *D. immitis* and *Brugia pahangi* infect dogs, a specific diagnosis cannot be obtained due to the conserved nature of nematode miRNAs miR-71 and miR-34 (96). Given their role in longevity (70), it would not be surprising if they are conserved among various nematodes. Studies attempting to identify stage and sex-specific miRNAs in *D. immitis* reveal that the abundant miRNAs among the microfilaria (mf), L3, L4, and adult *D. immitis* show temporal regulation, but stage-specific or sex-specific miRNAs could not be identified (97). The biomass of these parasites during the early stages of infection, the duration for which a particular developmental stage is present in the host before it undergoes molting, and the half-life of the miRNA are some of the factors to keep in mind while undertaking such studies. Perhaps, looking into miRNA panels instead of individual miRNAs could address the specificity issues in a diagnostics development.

## Toxocara canis

The members of the genus *Toxocara* infecting dogs and cats have a worldwide prevalence. The zoonotic condition, toxocariasis in humans, results from migrating larvae that may be present in the body tissues, eyes, or brain. Toxocariasis has a global seroprevalence of 19% (99), but it highly varies depending on the geographical location. Antibodies to *Toxocara* have also been detected in pregnant women and animal hoarders (100, 101). Considering recent research that implies a correlation between neural toxocariasis and neurological dysfunction and cognitive impairment (102), the identification of miRNAs in *Toxocara* could help uncover underlying molecular mechanisms and provide novel diagnostic or therapeutic targets. In 2008, a nucleotide sequence complementary to *C. elegans* miRNA, lin-4, was identified in the 3′ UTR of at least four abundant novel transcripts of *Toxocara* with seemingly inhibitory properties toward transcription and translation. It was only in 2016 that the first exploration of miRNAs of *Toxocara canis* was conducted, and the adult male and female-derived miRNAs were cataloged (103). A prediction of miRNAs participating in host-parasite interactions, along with the identification of secretory miRNAs, miR-71, miR-34, and miR-100, suggests their highly conserved roles. Approximately 35 miRNAs targeting the parasite's ABC transporter, cytochrome P450, and MDR-associated genes were identified, which could potentially regulate their transcription in response to anthelmintic presence. Such miRNAs could be leveraged for drug response studies. The current literature shows a couple of studies (104, 105) investigating miR-21 and miR-103a as diagnostic biomarkers for toxocariasis. However, the parasite origin of these miRNAs is debatable since they were also found in the sera of uninfected/control individuals. Other studies have looked at changes in the host-derived miRNAs from specific tissues, such as the lung, liver, and spleen, in response to migrating *T. canis* (106–108). Limited studies have been undertaken to explore miRNAs of *T. canis*. The veterinary community understands that 90% of new puppies acquire intestinal infections via transplacental or transmammary transmissions due to the reactivation of larvae from the tissues of the mother, probably as a response to pregnancy hormones. Research into miRNAs unique to reactivation or larval migration would be important to develop strategies to control or detect *Toxocara* infections in dogs and subclinical neglected zoonosis worldwide.

## *Ascaris* spp.

Members of the genus *Ascaris* are large roundworms that cause infections in humans and swine worldwide. The life cycle involves larval migration through the liver and lungs before maturing in the small intestine, which can cause fibrosis of the liver, respiratory signs, and intestinal blockage. MicroRNA research in *Ascaris* spp. showed apparent differences between miRNAs transcribed in adult male and female *Ascaris suum*, and among these were novel sex-specific miRNAs (57). Additionally, sex-specific miRNAs unique to the intestinal regions of the worm have also been detected (109). Although infecting two different host species, *A. lumbricoides* and *A. suum*, were proposed to represent a single species (110), and comparison of the miRNAs from the females of *A. lumbricoides* and *A. suum* supports this recent contention (111). MicroRNAs have been detected in EVs from all developmental stages of *A. suum* (55), such as the L3 and L4 larval stages and adults. With an understanding that miRNAs might play a role in host-parasite interaction and using miRNA target prediction tools, several *A. suum* miRNAs that potentially act to dampen host defense and prevent parasite elimination were predicted. Several components of the immune response, such as the complement system (*C1QA*), T-cell proliferation and activation (CD80, CD86, SLA-DOB), cytokines (IL-13 of T-helper cells, IL-25 and IL-33 of intestinal epithelial cells) and chemokine receptor (*XCR1*) appear to be targeted by *A. suum* miRNAs (55). Going a step further, a few abundant miRNAs and their targets have been experimentally validated. The miRNA, asu-miR-71-5p, was shown to interact with IFNGR1 and inhibit the host interferon response. This has been validated by direct binding of miR-71 to Irf4 mRNA using a luciferase assay (15). The miRNA asu-miR-791-3p can downregulate the mTOR pathway in murine primary CD4+ T cells *in vitro*. It can inhibit genes and pathways implicated in the differentiation of Th2 lymphocytes, including the interferon-gamma signaling pathway, the IL-2/STAT5 signaling pathway, and the mTOR signaling pathway itself. This downregulation disrupts the normal differentiation and function of Th2 lymphocytes and is associated with a protective adaptive immune response to parasitic infections (15). By targeting key genes, *A. suum*-derived miRNAs functionally disrupt several immune processes that can result in evasion and establishment of chronic infection. *Ascaris suum* miRNAs can modulate the host EV miRNA profiles, but studies to identify the *A. suum* miRNAs systemically from the infected host have not yet been successful. It could be due to the presence of adults in the small intestine and localized EV production, which may not go into the bloodstream in a detectable concentration (112).

## Haemonchus contortus

This is a blood-feeding trichostrongylid nematode residing in the abomasum of sheep and goats and is responsible for severe economic losses worldwide. Ingested as infective L3s in the pasture, the parasite can encyst as hypobiotic L4 stage before becoming an adult. MicroRNAs have been studied in all three developmental stages (L3, L4, and adults) (113) and from EVs and excretory-secretory products released by *H. contortus* (79). Interestingly, the source of miRNAs in the EVs from *H. contortus* L4 seems to be the gut, but not for EVs from adult worms (79). Evidence of *H. contortus* miRNA in the abomasum and regional lymph nodes suggests that the parasite might be using miRNA to communicate with the host or play a role in host-parasite interaction (79). For successful parasitism, it is important that parasite life cycle stages undergo a transition to the next life cycle stage at the right time in the right environment. The entire process may be tightly regulated with functional roles for miRNAs. A few miRNAs have been identified to show differential expression during this important stage transition from the pre-parasitic L3 stage to the parasitic L3 and L4 developmental stages. MicroRNAs such as hco-miR-34 and hco-miR-252 were suggested to have a role in stress response as the parasite undergoes the developmental transition from L3 to L4 (114). It would be equally beneficial for *H. contortus* to remain in a metabolically inactive L3 stage in the environment and transition to L4 within the host. Marks et al. identified two miRNAs, hco-miR-228 and hco-miR-235, in the *H. contortus* L3 stage that could prevent premature transition to L4. Sequences similar to those of hco-miR-228 have been identified in other nematodes, such as *B. malayi* and *A. suum* (https://www.mirbase.org/). A related miRNA, asu-miR-92 has been found to be most abundantly expressed in day 21-arrested L3 larvae of *A. suum* (115). MicroRNAs can be found clustered in the genome, and eight such clusters have been identified in *H. contortus* genome (113). One of the clusters, hco-miR-5352, was temporally expressed with relatively low levels in larvae and the highest levels in adult worms (79). Perez et al. (43) showed that a single secreted miRNA, hco-miR-5352 from *H. contortus*, can block IL-13–driven differentiation of epithelial secretory cells, such as tuft and goblet cells, in both mouse and sheep organoid models. By targeting key host regulators, including Klf4 and the IL-22 receptor subunit α1, and by interfering with Wnt and Notch signaling, hco-miR-5352 effectively promotes epithelial stem cell maintenance while dampening innate immune differentiation. This creates conditions that favor long-term parasite survival in the gut. Interestingly, hco-miR-5352 shares its seed sequence with the mammalian miR-92a family, hinting at a case of convergent evolution where the parasite has co-opted a host-like miRNA to manipulate immune and tissue-regenerative pathways to its own advantage (43). *Haemonchus contortus* belongs to clade V, and the homolog of hco-miR-5352 was identified in at least 10 other parasitic nematodes of medical and veterinary importance that belong to this clade.

Anthelmintic resistance in *H. contortus* and other trichostrongyles is a huge concern in the veterinary field, and there is growing evidence of differential miRNA expression in IVM-resistant vs. susceptible strains of *H. contortus* and *Teladorsagia circumcincta*. A particular miRNA, miR-9551, was significantly upregulated in resistant strains of these two nematodes and could be a potential biomarker for drug resistance in these nematodes (86). The selective release of miRNA from specific developmental stages of *H. contortus* reflects its adaptability to the environment and interaction with the host.

## *Brugia* spp.

*Brugia malayi* and *Brugia pahangi* are nematodes responsible for lymphatic filariasis in humans, with dogs suspected to serve as reservoirs of human infection (116, 117). Although dogs rarely exhibit clinical signs when infected, their role as reservoir hosts raises concerns, particularly in areas with significant human-animal interactions (118). The lymphatic filariasis caused by these parasites involves complex immune modulation, allowing them to evade host defenses and establish chronic infections. *Brugia* spp. miRNAs have been studied in adult males and females, microfilaria, and the infective larvae (56, 119), and exosome-like vesicles derived from the microfilariae (80, 120). Stage-specific expression of miRNA has been well-defined, with bma-miR-71 being 5–7 times more highly expressed in microfilariae than adults (119) and bpa-miR-5364 showing a 12-fold increased level as the parasite transitioned from mosquito vector to the mammalian host (56). This study identified bpa-miR-5364 as being specific to clade III nematodes. Functional assays to determine miRNA activity have been established in *Brugia* spp. (76) and the interaction between bpa-miR-5364 and its three potential targets was confirmed by dual luciferase assay (56).

Recent studies on *B. malayi* identified highly abundant miRNAs such as bma-miR-71, known to be involved in modulating immune responses (76, 119). This miRNA targets components of the IGF-1/insulin-like signaling pathway, including AGE-1, PDK-1, and AKT-1, which are the key determinants of longevity, stress resistance, and neuronal development (70). Moreover, bma-miR-71 affects DNA damage checkpoint pathways by modulating the expression of CHK-1 and CDC-25 1, and CDC-25.2 (70). Through these pathways, bma-miR-71 enhances the forkhead transcription factor DAF-16 activity, a key regulator of antioxidant, antimicrobial, and metabolic enzymes, leading to extended parasite lifespan and stress resistance (121). Additionally, bma-miR-71 was found to be loaded into EVs and internalized by immune cells, and was shown to regulate the levels of nitric oxide and the expression of RNA-induced silencing complex (RISC) components and host miRNAs that are associated with immune responses (122). This regulation could allow the parasite to evade immune detection and establish a chronic infection.

*Brugia malayi* infection induces the overexpression of miRNAs such as mmu-miR-125 b-5p, mmu-miR-146a-5p, and mmu-miR-378-3p in mouse macrophages, where the prefix “mmu” denotes *Mus musculus*, indicating that these findings were obtained from a murine model of infection. These miRNAs promote macrophage activation, increase inflammation, and cell-to-cell communication (123, 124). Most notably, mmu-miR-125b-5p can activate macrophages to express more stimulatory molecules, such as CD80 and interferon-gamma (IFNγ), key immune response molecules (123). Narasimhan et al. demonstrated that *B. malayi* microfilaria can manipulate the host immune response by interfering with the mechanistic target of rapamycin (mTOR) signaling pathway. The mTOR pathway plays an important role in the metabolic regulation of immune cells, balancing catabolic and anabolic processes necessary for cellular function and effective immune response. Microfilariae can influence the metabolism of host cells by inhibiting the phosphorylation of mTOR and the downstream effectors 4EBP1 and p70S6K. A similar reduction in the phosphorylation of mTOR was observed when mf-derived EVs were incubated with human monocytes (80). Notably, these EVs contained miRNAs, mainly miR-100, miR-7, let-7, miR-71, miR-4299, miR-34, miR-9, miR-92, miR-31, and miR-99, which can target genes related to the mTOR signaling pathway (80). Thus, miRNAs in the EVs appear to participate in modulating the host immune response.

## Strongyloides stercoralis

*Strongyloides stercoralis* can affect dogs by causing diarrhea, malnutrition, and respiratory complications, especially in puppies and immunocompromised animals, because the parasite can reinfect and reproduce within the same animal. Furthermore, *S. stercoralis* has zoonotic potential, posing a risk of transmission to humans. Until now, miRNA profiles of parasitic adult females, infective L3 (iL3), and L1 stages of *S. stercoralis* have been established (125, 126). Similar to other parasitic nematodes, conserved, shared, and novel miRNAs specific to the parasite species and to the developmental stages were discovered, highlighting the distinct roles miRNAs play in *S. stercoralis* infection and reproductive activities. A comparison with other species within this genus, *S. ratti* and *S. papillosus*, showed significant distinctions in the expression of miRNAs. Infective L3s used by Pomari et al. were isolated from human stools, whereas those used by Peixi et al. were from the feces of an experimentally infected dog. In the future, the two datasets provide a unique opportunity to compare iL3 miRNA catalogs to determine if the host influences the miRNA expression.

## Conclusions

The last decade has witnessed an increasing interest in miRNA-mediated gene regulation, highlighting the multifaceted impact of these potent regulatory molecules. Parasitic nematodes have a remarkable capacity to influence their host's immune response through intricate miRNA-based mechanisms to facilitate parasite persistence and tolerance. Moreover, the differential expression of miRNAs at different developmental stages in the parasite life cycle further confirms that they play a critical role in the development and survival of parasites. Despite these advances, there are several significant challenges in miRNA research on parasitic nematodes. Currently, the predictions of miRNA targets are bioinformatics-based, and as these tools were designed for human or model organisms, they poorly represent species-specific miRNA–mRNA interactions in parasitic nematodes. The precise identification of miRNA targets should be a priority area that would significantly enhance our understanding of parasitic nematode infection mechanisms. Additionally, the lack of intact genomic information in many parasitic nematode species, particularly the 3′ UTR where most canonical miRNAs reside, further impedes a detailed investigation of these interactions. Unfortunately, little experimental work has been done to investigate the functions of parasite miRNAs. However, the increasing amount of genome, transcriptome, and proteome data will aid in characterizing the roles of miRNAs in parasitic infection and biology. While *in vitro* and *in silico* studies are valuable, they do not always fully recapitulate the complex dynamic microenvironment of infection. In this context, the use of *C. elegans* as a model serves as a powerful system for studying miRNA function, owing to its well-annotated genome and availability of extensive genetic tools. The emergence of organoid culture systems like human-derived intestinal organoids (HIOs) represents a powerful tool to study host-parasite interactions under physiologically relevant conditions. These systems allow longer *in vitro* survival, developmental processes, and interactions with immune cells to generate a more functional snapshot of parasite miRNAs. For instance, *Anisakis* EVs have proven to be capable of modulating gene expression in HIOs by affecting cell cycle regulation, apoptosis, and immune pathways (127). These models give a functional overview of parasite miRNAs in active stages, showing how they control host gene expression and epithelial responses that are difficult to observe in conventional *in vitro* models.

Although miRNAs are potential diagnostic biomarkers for certain parasitic diseases, their limitations must be acknowledged regarding specificity, sensitivity, and the complexity of their interactions. Standardization of methodologies and procedural details is required to overcome inconsistencies in research outcomes, allowing accurate comparison of results between different research groups. Overcoming these limitations will be crucial for translating miRNA research into practical diagnostic tools and therapeutic strategies. Understanding and harnessing the regulatory power of miRNAs in parasitic nematodes holds great promise for developing new diagnostic tools, therapeutic strategies, and effective control measures against these parasites. As research continues to evolve and these strategies are refined and expanded upon, there is a distinct possibility of miRNA-related research revolutionizing the field of parasitic nematode infection control.
